# Predictors of Vitamin D Status in Religious and Intermittent Fasting: A Comparative Study in Orthodox Nuns and Women from the General Population

**DOI:** 10.3390/nu17101656

**Published:** 2025-05-13

**Authors:** Spyridon N. Karras, Konstantinos Michalakis, Maria Kypraiou, Antonios Vlastos, Marios Anemoulis, Georgios Koukoulis, Zadalla Mouslech, Filotas Talidis, Costas Haitoglou, Georgios Michos, Evangelos G. Papanikolaou, Dimitrios Skoutas, Neoklis Georgopoulos, Georgios Tzimagiorgis

**Affiliations:** 1 Laboratory of Biological Chemistry, Medical School, Aristotle University, 54124 Thessaloniki, Greece; haitoglu@auth.gr (C.H.); tzimagio@auth.gr (G.T.); 2 Department of Obesity and Metabolism, 11521 Athens, Greece; kostismichalakis@hotmail.com; 3 Assisting Nature Centre of Reproduction and Genetics, 57001 Thessaloniki, Greece; mariabioanalysis@yahoo.gr (M.K.); papanikolaou@assistingnature.gr (E.G.P.); 4 Medical School, Aristotle University, 54124 Thessaloniki, Greece; antonisvlastos1958@gmail.com (A.V.); mariosanemoulis@hotmail.com (M.A.); 5 Department of Endocrinology, University of Thessaly, 41500 Larissa, Greece; gnkouk@uth.gr; 6 1st Department of Internal Medicine, AHEPA Hospital, 54636 Thessaloniki, Greece; zmouslech@gmail.com; 7 Endocrine Practice, 52100 Kastoria, Greece; talidisf@gmail.com; 8 Third Department of Obstetrics and Gynecology, Aristotle University, 54124 Thessaloniki, Greece; giomichos@hotmail.com; 9 Thermi Clinic, 57001 Thessaloniki, Greece; skoutasd@otenet.gr; 10 Division of Endocrinology, University of Patras, 26504 Patras, Greece; neoklisgeorgo@gmail.com

**Keywords:** vitamin D, 25-hydroxyvitamin D, Orthodox monasticism, intermittent fasting, lifestyle factors, sun exposure, visceral fat, religious practices, parathyroid hormone, nutritional epidemiology

## Abstract

**Background:** Vitamin D plays a key role in bone metabolism and immune regulation. Populations with restricted sun exposure or limited dietary intake are particularly vulnerable to vitamin D deficiency. Orthodox Christian nuns represent a unique group in this regard, due to traditional clothing, limited outdoor activity, and prolonged religious fasting. However, few studies have compared them with lay individuals following similar dietary practices. **Objective:** This study aimed to investigate predictors of serum 25-hydroxyvitamin D [25(OH)D] concentrations in two female populations: Orthodox Christian nuns and women from the general population practicing intermittent (religious or non-religious) fasting. We also aimed to develop predictive models of vitamin D status for each group based on lifestyle and biochemical parameters. **Methods:** A total of 85 women (40 Orthodox nuns and 45 laywomen), aged 30–50 years, were enrolled. Serum 25(OH)D, parathyroid hormone (PTH), calcium levels, and anthropometric indices, including body mass index (BMI), total body fat, and visceral fat, were measured. Dietary calcium and vitamin D intake, as well as sun exposure, were assessed using validated questionnaires. Separate stepwise multiple regression models were constructed for each group to identify independent predictors of 25(OH)D concentrations. An additional combined model, including all participants, was also explored. **Results:** PTH was the most significant predictor, negatively correlating with 25(OH)D concentrations in both groups (*p* = 0.038), highlighting its regulatory role in vitamin D metabolism. When analyzed separately, the model for Orthodox nuns showed stronger explanatory power (adjusted R^2^ = 0.718; *p* = 0.013) compared with the control group (adjusted R^2^ = 0.362; *p* = 0.038), with PTH emerging as a key predictor in both. **Conclusions:** Distinct predictors of vitamin D status were identified in each group, reflecting the complex interplay between lifestyle and physiological factors. These findings suggest that targeted interventions, such as addressing PTH regulation in fasting populations or enhancing sun exposure in the general population, may be more effective in preventing vitamin D deficiency depending on the context.

## 1. Introduction

Vitamin D is essential for regulating bone and calcium physiology, but also in various extra-skeletal processes, such as immune modulation, metabolic regulation, and cell proliferation [1,2]. In addition to its classical role in skeletal health and calcium phosphate homeostasis, vitamin D is now recognized as a pleiotropic hormone involved in numerous extra-skeletal processes. These include the modulation of the innate and adaptive immune responses, the regulation of insulin secretion and sensitivity, influencing cell proliferation and differentiation, and potential effects on cardiovascular and neurocognitive function. Despite its significance, vitamin D deficiency remains highly prevalent worldwide, particularly in populations with limited sun exposure and specific cultural or religious practices and sartorial habits [3,4]. Among these groups, Christian Orthodox monasteries present a unique case. They follow a secluded lifestyle, adhere to traditional dress codes that limit skin exposure, and engage in extended periods of religious Orthodox fasting, often lasting over 180 days annually [5].

Several observational studies have suggested that restrictive diets, particularly those avoiding dairy and eggs, may lead to lower serum 25-hydroxyvitamin D [25(OH)D] concentrations, potentially increasing the risk of deficiency [6,7]. Furthermore, reduced sunlight exposure due to clothing or an indoor lifestyle has been associated with impaired vitamin D status across various populations [8]. However, the relative contribution of these factors in specific religious or culturally unique subgroups remains unclear. These lifestyle factors, individually and in combination, are likely to affect vitamin D status and present an adverse outcome of this vital subset of the Mediterranean diet, as we previously described [5,6,7,8]. Nevertheless, data on predictors of vitamin D status in these populations with unique lifestyles and dietary patterns are scarce, particularly regarding direct comparisons with women from the general population.

In addition to highlighting potential physiological variations in vitamin D status within populations from the same geographical region and similar dietary practices, predictive models could guide the development of tailored strategies for preventing and managing vitamin D deficiency in culturally diverse populations. This approach could be of particular importance in countries like Greece, where vitamin D food fortification policies are absent, and vitamin D deficiency rates are similar or even higher compared with those in higher latitudes and countries where food fortification of dairy products is adopted for the general population, since decades [5,6,7,8].

In this study, we aimed to investigate the determinants of vitamin D status in two distinct female populations: Orthodox Christian nuns practicing religious Orthodox fasting (a subtype of intermittent fasting throughout the year), and women from the general population practicing intermittent fasting, either for religious or non-religious reasons. While both groups incorporate fasting behaviors, only the monastic group adheres to a traditional Orthodox fasting pattern. In contrast, the general population group followed a time-restricted eating regimen of intermittent fasting without dietary exclusions, maintaining a regular intake of protein and animal products. Their overall lifestyle, physical activity levels, and sun exposure patterns also differed significantly. We assessed a range of potential predictors of serum 25(OH)D concentrations, including body composition (BMI and visceral fat), parathyroid hormone (PTH), calcium levels, dietary intake, and sun exposure. Moreover, we sought to construct population-specific predictive models to identify which factors most strongly influenced vitamin D status in each group, with a discourse on current supplementation strategies and future research agenda. While multiple studies have explored biochemical predictors of vitamin D status, such as PTH, calcium, and phosphate, our study is, to our knowledge, the first to directly compare these relationships in a culturally and religiously distinct population—Orthodox Christian nuns—versus age-matched women from the general population practicing non-religious intermittent fasting.

## 2. Methods

### 2.1. Design

This was a cross-sectional comparative study conducted between September 2023 and March 2024 in the central parts of Northern Greece in two female premenopausal populations.

### 2.2. Study Populations

We recruited a total of 85 adult women aged 30–50 years, comprising two groups: 40 Orthodox Christian nuns residing in monasteries, and 45 women from the general population practicing intermittent (non-religious) fasting. The study protocol followed the same ethical and methodological standards described in our previous research [5,6,7,8] and received approval from the Aristotle University Bioethics Committee in Thessaloniki, Greece. The inclusion criteria included female sex, aged between 30 and 50 years, absence of chronic diseases affecting vitamin D metabolism, and no use of vitamin D or calcium supplementation in the previous 6 months. The exclusion criteria included the presence of chronic diseases known to affect vitamin D metabolism, such as chronic kidney disease (stage ≥ 3), liver cirrhosis, malabsorption syndromes (e.g., inflammatory bowel disease and celiac disease), or primary hyperparathyroidism. Participants taking medications that influence bone or vitamin D status—including corticosteroids, anticonvulsants, bisphosphonates, vitamin D analogs, or hormone replacement therapy—were also excluded.

### 2.3. Dietary Regimens

Orthodox nuns followed the Athonian type of fasting, as previously described [5,6,7,8,9].

In brief, this pattern includes an 8 h feeding window (approximately between 07:00 and 15:00), aligning with traditional monastic practice and long fasting intervals between meals.

At the end of this study, participants’ adherence to the prescribed dietary protocol was assessed through a 3-day food diary. Nutrient intake was analyzed using the Food Processor Nutrition Analysis Software (version 2021, ESHA Research, Salem, OR, USA; accessed 2 August 2024). General-population fasters adopted a 16:8 dietary pattern, with an 8 h feeding time frame from 11.00–19.00, without avoidance of animal products and particular sartorial or religious habits.

### 2.4. Anthropometric and Biochemical Assessment

All participants underwent standardized anthropometric and biochemical assessments, as previously described [5,6,7,8]. Body weight, height, and BMI were recorded [10]. Body weight was measured to the nearest 0.01 kg using a calibrated digital scale (K-Tron P1-SR, Onrion LLC, Bergenfield, NJ, USA), with participants barefoot and wearing light clothing. Body mass index (BMI) was then computed by dividing weight (kg) by height squared (m^2^). Total and visceral body fat were assessed by bioelectrical impedance analysis (BIA) [X] (SC-330 S, Tanita Corporation, Tokyo, Japan) [11,12].

Fasting blood samples were collected in the morning, and serum concentrations of 25-hydroxyvitamin D [25(OH)D], intact parathyroid hormone (PTH), insulin, total calcium, phosphorus, and albumin were measured. Fasting blood samples were also analyzed for serum glucose (mg/dL) and insulin (μIU/mL) concentrations using standard enzymatic and immunoassay methods, respectively. These parameters were included to assess potential associations with vitamin D status and were used in exploratory regression models. Vitamin D was quantified using enzyme-linked immunosorbent assay (ELISA) [13]. Serum calcium levels were assessed using the COBAS8000 automated analyzer (Roche Diagnostics GmbH, Mannheim, Germany). Measurements of parathyroid hormone (PTH), 25-hydroxyvitamin D [25(OH)D], and insulin were performed on the COBAS e 602 module via electro-chemiluminescence (ECL) assay. The reference values and inter-/intra-assay coefficients of variation were as follows: 8.4–10.2 mg/dL of calcium (intra: 0.5–1.3%; inter: 0.8–1.3%), 15–65 pg/mL of PTH (1.6–6.9 pmol/L; intra: 2.5–3.4%; inter: 1.1–2.0%), 25(OH)D ≥ 30 ng/mL (intra: 3.4–13.1%; inter: 2.2–6.8%), and 5–20 IU/mL of insulin (CV: 3.2–6.2%).

### 2.5. Dietary Intake and Sun Exposure

Dietary calcium and vitamin D intake were assessed using a validated food frequency questionnaire (FFQ) [9,14], adapted for the Greek population (Supplementary Materials)**.** Information on the consumption of vitamin D-rich foods and calcium was collected and analyzed. Weekly sun exposure was estimated with a semi-structured questionnaire capturing the duration and percentage of body surface area exposed, as described previously (Supplementary Materials) [15]. All questionnaires and interviews were conducted in person by trained personnel, including a registered dietitian and a physician with expertise in endocrinology and clinical nutrition. Both researchers followed standardized procedures and were experienced in administering validated lifestyle and dietary questionnaires in population-based studies.

### 2.6. Statistical Analysis

A series of multiple linear regression models were constructed to explore determinants of serum 25(OH)D concentrations in both groups. Specifically, we used the following:

- Model 1: Demographic and anthropometric parameters (age, BMI, calcium and vitamin D intake, and sun exposure);
- Model 2: Model 1 plus total and visceral fat, as obtained by BIA analysis;
- Model 3: Μodel 2 plus PTH and insulin concentrations.

Continuous variables were summarized using either mean ± standard deviation (SD) values or median and interquartile range (IQR) values, based on their distributional characteristics. Differences between groups were tested using independent-samples t-tests or Mann–Whitney U tests. Categorical variables were compared using chi-square tests. Statistical significance was set at *p* < 0.05. All analyses were performed using SPSS v25.0 (IBM Corp, Armonk, NY, USA).

## 3. Results

Table 1 presents the demographic, anthropometric, and biochemical characteristics of both groups. The two groups were comparable for all baseline characteristics with the exception of the higher dietary calcium intake for women of the general population and 25(OH)D and PTH concentrations, where Orthodox nuns had a non-significantly lower vitamin D status (22.5 ± 8.7 ng/mL) compared with the general population (26.4 ± 9.1 ng/mL) and significantly higher PTH concentrations (42.0 ± 15.1 pg/mL) compared with the control group (24.6 ± 8.2 pg/mL, *p* < 0.01). The mean duration of monastic life for Orthodox nuns was 18.6 years, with a range from 0.25 to 59 years.

Models 1 and 2, including demographic and anthropometric factors (age, BMI, calcium and vitamin D intake, total sun exposure, and visceral fat), showed limited predictive value. None of the variables reached statistical significance, and the models explained a modest proportion of the variability in vitamin D concentrations (R^2^ ≈ 18–35%) for both groups. Model 1 explained only 18–24% of the variation in vitamin D concentrations (R^2^ = 0.184; *p* = 0.671), with no statistically significant predictors (Table 2). Model 2 increased the explanatory power (32–36%) (R^2^ = 0.362; *p* = 0.480), with stronger but non-significant trends for both groups (Table 3).

**Figure 1:**
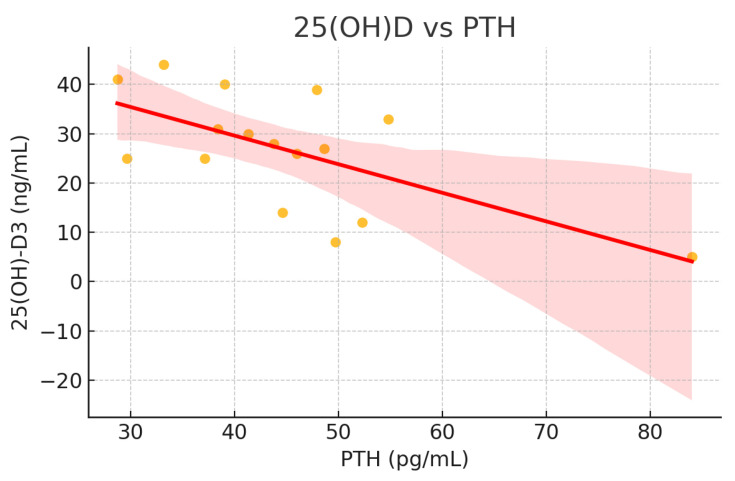
Scatterplot of serum 25(OH)D levels against PTH (pg/mL) for both groups.

**Figure 2:**
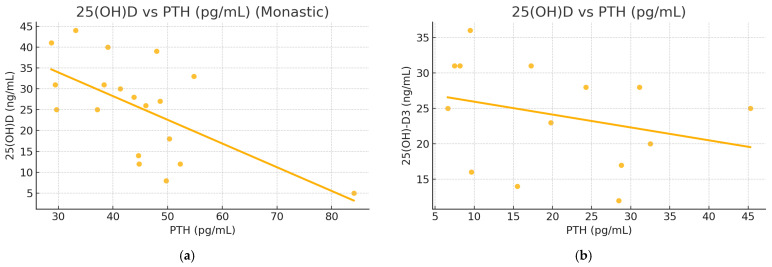
Scatterplot of serum 25(OH)D concentrations against PTH for monastic (**a**) and general population groups (**b**).

**Figure 3:**
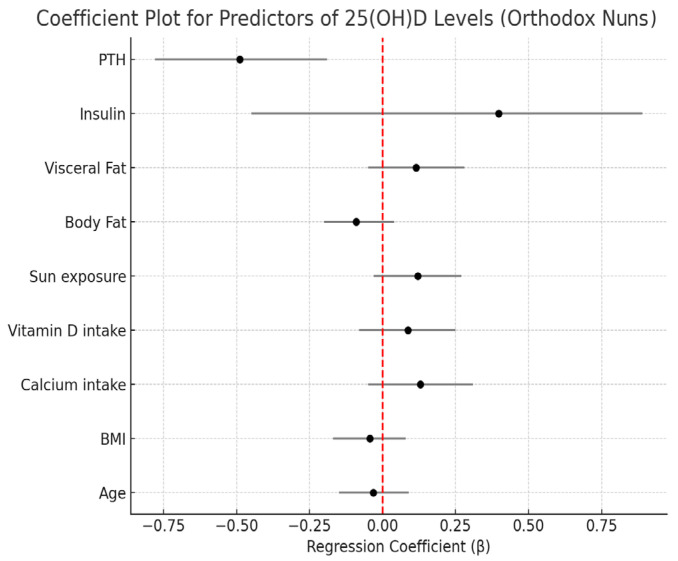
Coefficient plot for predictors of 25(OH)D levels in Orthodox nuns.

Model 3 substantially improved the explanatory power of the variability in 25 (OH)D concentrations, and PTH emerged as the most significant predictor, showing a negative association with 25(OH)D concentrations for both groups (*p* = 0.038) (Table 4) (Figure 1). Insulin showed a positive but non-significant trend (*p* = 0.266). When studied separately, Orthodox nuns had a stronger regression model (R^2^ = 0.718; *p* = 0.013) compared with the control group (R^2^ = 0.362; *p* = 0.038), identifying PTH as a significant predictor and explaining 72% of the variability in 25(OH)D concentrations compared with 36.2% for the general population group (Figure 2 and Figure 3). Serum calcium and phosphate were not statistically significant predictors of 25(OH)D in any model.

## 4. Discussion

This study aimed to investigate factors influencing vitamin D status (25(OH)D) in two distinct population groups. The results highlight population-specific relationships between markers of calcium homeostasis, environmental factors, such as sun exposure, dietary intake, and vitamin D concentrations. The analysis of vitamin D status in Orthodox Christian nuns and women from the general population practicing intermittent fasting yielded insightful findings on the complex interplay of lifestyle and physiological factors in determining serum 25(OH)D concentrations.

A high prevalence of hypovitaminosis D in similar monastic populations, primarily due to limited sun exposure resulting from religious sartorial habits and dietary practices associated with Orthodox fasting (which restricts certain food groups for extended periods), has been previously reported [16,17,18,19,20]. In the case of Orthodox nuns, sun exposure is restricted not only by limited outdoor activity but also by the minimal skin surface exposed to sunlight, due to the traditional religious attire that covers most of the body throughout the year.

Despite greater access to outdoor activities and dairy products, laywomen demonstrated comparable vitamin D status, challenging the assumption that adequate sun exposure and a balanced diet are pivotal factors in maintaining optimal vitamin D levels in the general population [3,4]. Although both groups followed similar dietary patterns of intermittent fasting, with the exclusion of animal products in Orthodox nuns, our results suggest that additional physiological factors may also affect vitamin D equilibrium [1,2]. Further studies are necessary to explore the genetic and long-term effects of various biological regulators on this intriguing interplay.

A consistent inverse relationship between serum 25-hydroxyvitamin D [25(OH)D] and PTH has been previously documented, suggesting that PTH suppression remains a reliable, albeit nuanced, surrogate marker of vitamin D sufficiency [1,2,3,4]. Interestingly, in our study, both groups showed a negative association between PTH and 25(OH)D, reaffirming the regulatory role of PTH in vitamin D metabolism. However, the monastic group demonstrated a stronger influence of PTH on 25(OH)D status. Although 1,25-dihydroxyvitamin D [1,25(OH)_2_D] is the hormonally active form of vitamin D, its measurement is not routinely used to assess vitamin D status. Serum levels of 1,25(OH)_2_D are short-lived, subject to tight homeostatic control, and often remain normal or elevated in early deficiency states due to compensatory increases in PTH. In contrast, 25(OH)D is more stable, reflects endogenous production and intake, and is the preferred biomarker in population-based studies and clinical practice. In addition, the inclusion of fasting glucose and insulin measurements was based on growing evidence that vitamin D may influence glucose homeostasis. Vitamin D receptors are present in pancreatic β-cells and peripheral tissues, and vitamin D may enhance insulin secretion and improve insulin sensitivity [1,2,3,4]. Although insulin levels did not significantly predict 25(OH)D concentrations in our final models, their inclusion allowed us to explore possible metabolic pathways linking vitamin D with broader endocrine functions, particularly in premenopausal women.

To our knowledge, this is the first report on the predictive factors of 25(OH)D status in a female monastic community practicing Orthodox intermittent fasting, compared with an age-matched female population adopting a similar dietary pattern. These findings provide valuable insights into the complex factors regulating vitamin D metabolism in distinct population groups under widely adopted dietary habits, including intermittent fasting.

Our findings align with and extend the growing body of evidence supporting the complex, multifactorial nature of vitamin D metabolism, particularly its association with parathyroid hormone (PTH) dynamics across age, sex, and metabolic contexts [21,22,23,24,25]. Age has been identified as a critical modifier in this relationship. Previous studies have demonstrated that age-related declines in cutaneous vitamin D synthesis and renal hydroxylation capacity may result in diminished responsiveness to circulating 25(OH)D levels. Notably, older individuals exhibited higher PTH concentrations despite relatively adequate 25(OH)D status, possibly due to a form of vitamin D resistance. These age-dependent alterations underscore the limitations of fixed 25(OH)D cut-offs and argue for an individualized interpretation of vitamin D biomarkers, particularly in unique populations, like Orthodox monastic communities. In our study, this inverse association characterizing calcium homeostasis was evident despite significant differences in calcium intake and sun exposure between the two groups, reinforcing the biological interdependence of calcium-regulating hormones.

In this regard, the debate over the most informative marker of vitamin D status and its regulators has prompted investigations beyond total 25(OH)D, including the use of free and bioavailable fractions [26,27,28,29,30]. However, recent evidence suggests that while free and bioavailable 25(OH)D are theoretically more biologically relevant, their thresholds in predicting bone turnover or PTH responses are not consistently superior to those of total 25(OH)D [31,32]. Although novel markers such as VMR (the vitamin D metabolite ratio) and free/bioavailable 25(OH)D hold promise [33,34], current data favor total 25(OH)D in predicting endocrine and skeletal responses [1,2,3,4]. In Chinese women of childbearing age, PTH and bone turnover markers responded more robustly to changes in total 25(OH)D than to its free or albumin-bound forms [35,36]. Additionally, while serum 25(OH)D remains the cornerstone of vitamin D status evaluation, its interpretation should be contextualized within age, sex, season, geographic variability, individual metabolic profiles, and specific dietary patterns. Previous studies have demonstrated distinct seasonal oscillations in both 25(OH)D and PTH levels, with approximately two-fold differences in vitamin D status between summer and winter, further complicating diagnostic thresholds [37,38,39,40]. These findings support the incorporation of environmental and lifestyle factors—such as sun exposure and diet, especially in populations practicing Orthodox or other types of religious or non-religious intermittent fasting—into clinical assessments and public health strategies. Future large-scale, population-based longitudinal studies are needed to refine biomarker thresholds and improve the individualized approach to vitamin D supplementation strategies.

These preliminary data highlight the unique factors influencing vitamin D status in Orthodox Christian nuns and laywomen adopting intermittent fasting. Tailored interventions should be considered to prevent and manage vitamin D deficiency, especially in culturally unique subgroups like monastic communities, incorporating differences in dietary vitamin D and calcium intake, sun exposure, and specific dietary patterns. The finding that PTH was the strongest negative predictor of 25(OH)D in both groups aligns with its physiological role as a compensatory hormone in vitamin D deficiency. However, the higher explanatory power of the regression model among nuns (adjusted R^2^ = 0.72) suggests a more tightly regulated homeostatic system in fasting individuals, possibly due to more uniform dietary and lifestyle patterns. In contrast, the general population group exhibited a lower R^2^ (0.362), which may reflect greater inter-individual variability and the influence of unmeasured confounders, such as genetic factors or supplement use.

Although fasting insulin was included as a predictor, the HOMA-IR index would provide a more accurate reflection of insulin resistance. This was not calculated in the present study and should be considered in future analyses. In addition, alkaline phosphatase, a relevant marker of bone turnover and vitamin D deficiency, was not assessed in this study and represents another area for potential improvement in future research.

This study had several limitations, including its relatively small sample size. A small sample size could limit the generalizability and power of regression models, especially when building multiple predictors. In our study, we also did not check for multicollinearity or the normality of residuals. In addition, in the nuns’ group, the model based on stepwise regression explained a substantial proportion of the variability in PTH levels (R^2^ = 0.72). However, given that stepwise regression is a data-driven method, this high R^2^ may partially reflect overfitting to the sample data. We acknowledge that no external or internal validation (e.g., cross-validation or independent sample) was performed, which limits the generalizability of the model and may lead to an overestimation of its predictive power. Future studies including independent validation datasets are needed to confirm the stability of these associations. In contrast, the model for the laywomen group demonstrated a lower explanatory capacity (R^2^ = 0.362), indicating a weaker fit. This suggests that additional unmeasured variables—potentially including detailed dietary habits, sunlight exposure patterns, or genetic factors—may contribute to the regulation of PTH in this population. This limitation underscores the complexity of PTH regulation and the need for more comprehensive models in non-fasting populations.

Another important limitation of this study was the reliance on self-reported data for dietary intake and sun exposure, which may be subject to recall bias and misclassification. These biases could affect the accuracy of lifestyle-related variables, particularly in populations with structured or atypical daily routines, such as Orthodox nuns.

Although participants were matched for age and BMI, we did not control for other potential confounding variables such as occupation or socioeconomic status, which may have influenced lifestyle, diet, and sun exposure patterns. Additionally, while the questionnaires used were validated for the general Greek population, specific cultural adaptation for monastic life was not performed, which may have affected the precision of dietary and sun exposure reporting in the nuns’ group.

Another important limitation of the present study was its cross-sectional design, which precluded causal inference. Although low 25(OH)D is known to increase PTH secretion through physiological mechanisms, it is also possible that both vitamin D deficiency and elevated PTH levels are downstream effects of reduced sunlight exposure—a potential unmeasured confounder or mediator. Reverse causality cannot be ruled out, and further longitudinal studies or intervention trials are needed to clarify the temporal and causal relationships between sun exposure, vitamin D status, and PTH regulation.

The generalizability of our findings is limited by the population-specific context of this study. All participants resided in a single geographic region, and factors such as seasonality, latitude-related differences in UV exposure, and genetic variability affecting vitamin D metabolism were not accounted for. These unmeasured influences may impact vitamin D status and PTH regulation in other populations, including members of different religious orders, cultural practices, or those living in different climatic zones. Future studies incorporating these variables will be essential for the broader applicability of the results.

In resource-limited settings or large population-based studies where direct vitamin D testing may not be feasible, routine biochemical markers such as serum calcium and phosphate may offer useful, albeit indirect, insights into vitamin D status.

In our study, phosphate was not included in the regression models, despite its potential value as a low-cost surrogate marker in specific contexts. However, such associations should be interpreted cautiously, and further research is needed to validate their predictive utility in diverse populations. Insulin was included as a candidate predictor based on prior studies suggesting an association between vitamin D status and insulin resistance, particularly in premenopausal women. Although not retained in the final models, its inclusion allowed for a more comprehensive exploration of metabolic influences on 25(OH)D concentrations.

Finally, while serum 25(OH)D remains the definitive marker of vitamin D status, the inclusion of other biochemical parameters (e.g., PTH and calcium) in the regression models aimed to explore their physiological interplay with vitamin D. These markers may reflect adaptive responses to deficiency or provide indirect insights in settings where vitamin D testing is not accessible. However, we acknowledge that they are not substitutes for direct measurement, and future studies should prioritize direct assessment of 25(OH)D while considering these factors as complementary indicators rather than predictors.

Our findings support the previous literature regarding the inverse relationship between PTH and vitamin D status. However, the novelty of this study lies in the application of predictive modeling to two parallel populations with distinct religious and lifestyle characteristics, providing culturally specific insights into vitamin D metabolism.

While this study provides valuable insights into the factors affecting vitamin D levels in nuns and the general population, further research with larger sample sizes and more diverse populations is needed to confirm these findings and explore the genetic factors that may influence vitamin D metabolism. Moreover, longitudinal studies are required to better understand the long-term effects of lifestyle changes and supplementation on vitamin D status in similar populations.

## 5. Conclusions

In conclusion, this study highlights the complex factors influencing vitamin D levels in different populations. These findings suggest that tailored interventions are needed for groups with specific dietary and sartorial habits and provide potential insights into the complex factors regulating vitamin D metabolism.

## Figures and Tables

**Table 1:** Baseline characteristics of the study population.

Variable	Monastic Group (n = 40)	General Population (n = 45)	*p*-Value
Age (years)	52.4 ± 17.5	49.6 ± 14.3	0.314
Years in monastery (years)	18.6 ± 14.1	—	—
Calcium intake (mg/day)	467.5 ± 34	678 ± 69	0.034
Vitamin D intake (IU/day)	245 ± 11	267 ± 18	0.23
Sunshine exposure (hours/week)	4.23 ± 1.2	11.2 ± 3.8	<0.01
BMI	27.3 ± 4.9	25.9 ± 3.8	0.151
Body fat (%)	36.0 ± 7.8	34.1 ± 6.1	0.220
Visceral fat	7.7 ± 3.9	6.1 ± 2.7	0.031
Insulin (μIU/mL)	11.3 ± 10.6	8.4 ± 6.9	0.081
PTH (pg/mL)	42.0 ± 15.1	24.6 ± 8.2	<0.01
25(OH)D (ng/mL)	22.5 ± 8.7	26.4 ± 9.1	0.062
Glucose (mg/dL)	92.4 ± 9.5	89.8 ± 8.7	0.129
Calcium (mg/dL)	9.1 ± 0.3	9.4 ± 0.3	0.914
Phosphate (mg/dL)	3.7 ± 0.5	3.9 ± 0.4	0.087

Values are presented as means ± standard deviations (SDs). Predictive models.

**Table 2:** Predictive Model 1 for determinants of 25 (OH)D concentrations.

Predictor	Coefficient	*p*-Value
Age (years)	0.064	0.665
BMI	3.015	0.210
Calcium intake (mg/d)	0.980	0.435
Vitamin D intake (IU/d)	0.142	0.123
Sunshine exposure (h/w)	1.381	0.171
Calcium (mg/dL)	0.054	0.779
Phosphate (mg/dL)	−0.217	0.528

**Table 3:** Predictive Model 2 for determinants of 25 (OH)D concentrations.

Predictor	Coefficient	*p*-Value
Age (years)	−0.726	0.215
BMI	−0.055	0.985
Calcium intake (mg/d)	0.812	0.061
Vitamin D intake (IU/d)	0.143	0.056
Sunshine exposure (h/w)	0.109	0.064
Calcium (mg/dL)	0.123	0.708
Phosphate (mg/dL)	−0.278	0.412
Body fat (%)	−1.571	0.101
Visceral fat	7.495	0.140

**Table 4:** Final stepwise regression model (Model 3) for predicting serum 25(OH)D concentrations in Orthodox nuns and general population women. Model 3 includes demographic, anthropometric, and biochemical variables.

Predictor	Orthodox Nuns β (*p*-Value)	General Population β (*p*-Value)
Age (years)	−0.032 (0.784)	−0.017 (0.852)
BMI	−0.044 (0.725)	−0.039 (0.791)
Calcium intake (mg/day)	0.129 (0.214)	0.119 (0.178)
Vitamin D intake (IU/day)	0.086 (0.341)	0.101 (0.229)
Sun exposure (h/week)	0.121 (0.261)	0.144 (0.191)
Calcium (mg/dL)	0.045 (0.737)	0.061 (0.698)
Phosphate (mg/dL)	−0.084 (0.611)	−0.112 (0.493)
Body fat (%)	−0.091 (0.438)	−0.073 (0.502)
Visceral fat	0.115 (0.301)	0.132 (0.227)
Insulin (μIU/mL)	0.398 (0.422)	0.790 (0.231)
PTH (pg/mL)	−0.489 (0.013)	−0.549 (0.038)

Adjusted R^2^: 0.718 for nuns; 0.362 for general population.

## Data Availability

The original contributions presented in this study are included in this article/Supplementary Material. Further inquiries can be directed to the corresponding author.

## Supplementary Materials

The following supporting information can be downloaded at https://www.mdpi.com/article/10.3390/nu17101656/s1. Supplementary File S1: Dietary intake questionnaire (vitamin D and calcium-focused FFQ); Supplementary File S2: Sun exposure questionnaire.
