# Supplementary material for: Predictors of Vitamin D Status in Religious and Intermittent Fasting: A Comparative Study in Orthodox Nuns and Women from the General Population
Source: Nutrients. 2025 May 13;17(10):1656. doi: 10.3390/nu17101656 (PMC12114524; doi:10.3390/nu17101656)
Supplement: Supplementary file 1 [file nutrients-17-01656-s001.zip › nutrients-3634321-supplementary.pdf]

Supplementary File S1: Dietary Intake Questionnaire (Vitamin D and Calcium Focused FFQ)

This questionnaire is designed to assess dietary intake of vitamin D and calcium over the past month. Participants are asked to indicate the frequency with which they consumed the following foods.

Section A: Dairy and Fortified Products
1. Milk (cow, goat, or fortified plant-based) - glasses per day/week/month:
2. Yogurt - servings per day/week/month:
3. Cheese (any type) - servings per day/week/month:
4. Fortified breakfast cereals - servings per week:
5. Fortified margarine or spreads - frequency:

Section B: Fish and Animal Products
6. Fatty fish (salmon, sardines, mackerel) - servings per week:
7. Cod liver oil or fish oil supplements - yes/no, dose:
8. Eggs - number per week:
9. Liver or organ meats - frequency per month:

Section C: Supplements
10. Vitamin D supplements - dose and frequency:
11. Calcium supplements - dose and frequency:

Section D: Other
12. Do you follow a vegetarian or vegan diet? Yes/No
13. Do you follow any fasting practices (e.g., religious)? Please describe:

Supplementary File S2: Sun Exposure Questionnaire

This questionnaire is designed to assess habitual sun exposure and potential cutaneous vitamin D synthesis.

Section A: Sun Exposure Habits

1. On average, how many hours per week do you spend outdoors between 10:00 am and 4:00 pm?
2. During which parts of the year are you usually outdoors? (e.g., spring, summer)
3. Which parts of your body are typically exposed when outdoors? (e.g., face, arms, legs)
4. Do you usually wear sunscreen? Yes/No. If yes, what SPF?
5. Do you avoid the sun (e.g., for religious, cultural, or medical reasons)? Yes/No

Section B: Clothing and Lifestyle

6. What type of clothing do you typically wear outdoors?
7. Do you work or spend leisure time indoors or outdoors?
8. Are you exposed to sunlight through windows at work or home?

Section C: Skin Type and Sensitivity

9. What is your skin type (I–VI)? (Fair to dark)
10. How easily do you tan or burn?
11. Do you have any diagnosed photosensitivity or skin conditions affecting sun exposure?

These questionnaires were adapted for use in the Greek population and reviewed for appropriateness among monastic and lay female groups. They are provided here to support transparency and replication of study methodology.
